# Chitosan nanoparticles used via different irrigation techniques: Syringe and laser activated irrigation via Er: YAG laser on smear layer and pushout bond strength: An In Vitro SEM assessment

**DOI:** 10.12669/pjms.41.11.12919

**Published:** 2025-11

**Authors:** Amer M. Alanazi, Kinza Bhutto, Muhammad Omar Niaz, Shaheryar Shafqat

**Affiliations:** 1 Amer M. AlanaziDepartment of Pharmaceutical Chemistry, Pharmaceutical Biotechnology Laboratory, College of Pharmacy, King Saud University, Riyadh 11451, Saudi Arabia; 2 Kinza Bhutto Department of Surgery, Aga Khan University, Karachi, Pakistan; 3 Muhammad Omar Niaz Department of Community and Preventive Dentistry, Army Medical College (National University of Medical Sciences), Rawalpindi, Pakistan; 4 Shaheryar Shafqat Graduate Research Assistant at the University of Memphis. USA

**Keywords:** Chitosan nanoparticles, Ethylenediaminetetraacetic acid, Laser-activated irrigation, Syringe irrigation

## Abstract

**Objectives:**

To quantify the efficacy of final canal disinfectants, ethylene-diamine-tetraacetic acid (EDTA) Chitosan nanoparticles (CHNPs) used via different irrigation techniques, i.e., Syringe irrigation (SI), laser-activated irrigation (LAI) on smear layer (SL), and push-out bond strength (POBS) of root canal filling material to dentin.

**Methodology:**

The present in vitro study was approved by the ethical committee of King Saud University and completed in three months (April 10 to July 10, 2025). Forty-four fully matured, single-rooted human mandibular premolars were selected. After confirming the working length, the root canal procedure was initiated, and the canal was shaped. The teeth were arbitrarily distributed into four distinct cohorts based on final disinfection protocols (n=11): Group-IA: (EDTA-SI), Group-IIA: (CHNPs+ SI), Group-IB: (EDTA-LAI), and Group-IIB: (CHNPs-LAI). Scanning Electron Microscopy (SEM) assessed SL removing efficiency. Root canal filling was performed on ten samples. The POBS and modes of failure were evaluated quantitatively with a universal testing machine and a stereomicroscope. ANOVA and Tukey post hoc test were used to analyze data, p<0.05.

**Results:**

The Cervical third of Group-IIB (NaOCl _+_ CHNPs-LAI) presented the maximum SL removal (12.36±0.56 MPa) and maximum POBS (12.36±0.56 MPa) of GFP. However, the apical section of Group-I (NaOCl _+_ EDTA-SI) samples revealed the lowest SL removal (3.55±0.42) and minimum POBS (6.43±0.42 MPa).

**Conclusion:**

Chitosan nanoparticles activated with laser as a final root canal disinfectant presented better SL removal and bond strength of AH plus sealer to the canal dentin.

## INTRODUCTION

Effective chemo-mechanical preparation is of paramount importance in endodontic therapy for the elimination of pulpal tissue and microbial biofilms. It has been observed that over 35% of the root canal surface remains uninstrumented, irrespective of the technique employed for preparing the canal.^1^ The primary endodontic irrigant solution employed in the field of endodontics is sodium hypochlorite (NaOCl) solution. However, NaOCl solution does not possess the capacity to remove the inorganic part of the SL, and its remnants impede the polymerization mechanism of the resin sealer.^2^

To address this deficiency, it is recommended to employ Ethylene-diamine-tetra-acetic acid (EDTA) after the disinfection of the canal using NaOCl. Its application is advocated due to its properties as a chelating agent, which facilitates the removal of the inorganic components of the SL.^3^ Nevertheless, prior literature has indicated that prolonged use of EDTA may compromise the structural integrity of the tooth, potentially resulting in dentin erosion.^4^ Therefore, researchers have sought to identify superior alternatives. Recently, chitosan has gained considerable attention within the healthcare sector. Chitosan is a naturally occurring polysaccharide that is obtained through the deacetylation process of chitin sourced from the exoskeletons of crustaceans and shrimps.^5^ Past studies conducted by Silva and coworkers have demonstrated that chitosan nanoparticles (CHNPs) exhibit chelating properties; thus, they possess the ability to solubilize the inorganic components of SL.^6^

In addition to the agents employed for canal disinfection, the technique in which they are administered also significantly impacts the efficacy of the SL removal and bond strength. The disinfection techniques may be classified into two main groups: manual delivery in the form of syringe irrigation (SI) and the utilization of machine-assisted agitation devices. Conventional SI is executed utilizing a syringe that exerts positive pressure. In SI approach, the irrigating solution is administered into the canal employing side-vented needles.^7^ It has been documented that in this technique, the solution extends only 1mm beyond the needle’s tip. In addition, the vapor lock effect may develop within the canals, which could interfere with the contact between the disinfectant and dentinal walls which further prevents it from penetration.^8^

Whereas Laser-Assisted Irrigation (LAI) utilizing Er: YAG laser demonstrates exceptional efficacy in the SL and debris elimination from the canal dentin. In this technique canal is subsequently filled with a disinfectant, and the laser tip is activated within the pulp chamber. The emission of laser pulses induces thermal effects in the fluid, resulting in the formation of bubbles within the irrigant^9^,^10^ The collapse of these bubbles generates shear flows capable of detaching SL and debris from the walls of the canal. Research conducted by Chakravarthy and colleagues revealed that the application of CHNPs and EDTA when activated by a diode laser yielded comparable outcomes in the elimination of SL.^11^

The current investigation was predicated on the supposition that no statistically significant difference exists in the eradication of SL and the POBS of root canal sealer disinfected with CHNPs when compared to the control EDTA, provided that a similar irrigation technique is employed. Additionally, it was also hypothesized that there would be no significant difference in SL elimination and POBS of the endodontic sealer when disinfection is carried out utilizing the LAI technique as opposed to the traditional SI. The research intends to investigate the impact of two distinct final root canal irrigants applied through different methodologies on SL elimination and POBS of root canal sealer concerning the canal dentin.

## METHODOLOGY

The present in vitro study was approved by the ethical committee of King Saud University. The study was completed in three months (April 4 to July 10, 2025). The present study follows checklist for reporting invitro study (CRIS) guidelines.

### Ethical statement:

The research experiments conducted in this article were approved by the Ethical Committee of King Saud University under IRB number: FC-19-2025, Dated: 1st March 2025.

### Samples preparation:

Forty-four adult human single-rooted premolars, possessing root length of 15 mm, were used in the study. The teeth included in the study were required to exhibit a moderate curvature ranging from 25° to 35° and to possess intact apices. Teeth exhibiting calcification, prior root canal treatment, cracks, fractures, or resorption were excluded. The samples included were meticulously debrided and cleaned using a hand scaler, subsequently preserved in a 0.1% thymol solution until the initiation of the experimental procedures. The crown portion of the included samples was bisected at the cementoenamel junction utilizing a slow-speed diamond disk with water cooling, preserving a root length of 15 mm.

### Root canal treatment:

Root canal procedure was executed employing manual #10 K-Files (MANI, Tochigi, Japan) to establish apical patency. The working lengths were reduced by 1 mm, making it 14 mm from the original length. Further preparation was conducted utilizing the crown-down technique with a rotary file (ProTaper Universal, Dentsply Maillefer) at 250 rpm, extending up to the F3 finishing file. Canal disinfection was performed continuously during the instrumentation process employing a 2.5% NaOCl solution. The canals were systematically classified into four distinct categories according to the final irrigant and technique employed (n=11 each).

### Group-IA: EDTA (SI):

The samples in this group were rinsed with 5 mL of 17% EDTA (Meta Biomed Co. Ltd, Korea) using a syringe equipped with a 27-gauge side-vented needle for 60 seconds as a final irrigant.

### Group-IB: CHNPs (SI):

In this group, a 0.2% CHNPs solution was employed as the final sterilizing agent for 60 secs by using a syringe with a 27-gauge side-vented needle. This formulation was accomplished by the dissolution of 0.2 grams of chitosan powder (NHI, Tangerang, Indonesia) in a 1% acetic acid solution, culminating in a total volume of 100ml.

### Group-IIA: EDTA (LAI):

The samples in this group were rinsed with 5 mL of 17% EDTA, which was activated using a laser. The activation was executed in PIPS (photon-induced photoacoustic streaming) mode employing an Er: YAG laser (Fotona, Ljubljana, Slovenia) at a wavelength of 2940 nm. Other laser parameters include a power output of 20 W and a frequency of 50 Hz. A 400 μm quartz tapered tip measuring 14 mm in length was placed on the coronal access opening of the chamber and was utilized with a pulse duration of 50 μs for a duration of 30 seconds. The water spray was deactivated. The irrigating solutions were continuously administered concurrently with the radiation. This protocol was executed twice, 30 seconds each, and culminated in one minute in total.^12^

### Group-IIB: CHNPs (LAI):

In this group, a 0.2% CHNPs solution was employed as the final sterilizing agent, which was activated using a laser using the same protocol as Group-IIA.

### Scanning electron microscope examination:

One specimen from each group underwent SEM analysis for SL removal assessment. Two longitudinal indentations were precisely marked on both the buccal and lingual surfaces of the roots. A chisel was subsequently employed to bisect the roots, and one-half of each specimen was selected for further examination. All preparatory procedures were conducted by a single operator to ensure consistency. The samples were affixed to aluminum stubs and were then sputter-coated with platinum for enhanced imaging. Thereafter, the root sections were observed under SEM (Hitachi S-4500, Tokyo, Japan) at diffrent magnification employing the Hülsmann^13^ grading system.

### Endodontic root filling procedure:

Subsequently, all canals were meticulously dried utilizing absorbent points (Puma Dent, China), followed by obturation employing the single cone technique with F3 gutta-percha (Dentsply Maillefer) that was marginally coated with AH Plus sealer (Dentsply DeTrey, Konstanz, Germany). The canal openings were subsequently filled with Cavit G (3M ESPE, Seefeld, Germany), and the teeth were maintained in an incubator at 37 °C and 100% humidity for a duration of seven days.

### Sectioning of obturated teeth:

The segmentation of the endodontically treated dentition was performed utilizing a slow-speed saw (Isomet 1000, Buehler, Lake Forest, IL) accompanied by continuous water cooling, to create discs of 1 mm thickness across the cervical, middle, and apical thirds of the root.

### POBS testing:

The POBS evaluations were conducted employing a universal testing machine (UTM) (Mechatronik, Feldkirchen-Westerham, Germany) at a crosshead speed of 1 mm per minute. A calibrated plunger was utilized to apply a vertical force to the filling material, featuring a tip diameter of 0.3 mm for the apical third and 0.5 mm for both the middle and cervical thirds. The force required to break the bond was measured in MegaPascals (MPa).

### Fracture pattern analysis:

Following the dislodgement of the root canal filling material from the canal, each specimen underwent examination utilizing a stereomicroscope (Olympus BX60, Japan) for failure mode assessment at 40x magnification. The mode of failure was evaluated independently by two calibrated and blinded assessors. The fracture patterns were characterized as adhesive, cohesive and admixed. Two independent reviewers assessed the fracture pattern Kappa reliability (0.91).

### Data analysis:

Data analysis was performed using SPSS 24.0 software (SPSS Inc., Chicago, IL, USA). The comparison among different tested groups was performed using one-way ANOVA post hoc Tukey analysis. (p<0.05).

## RESULTS

### SL removal assessment:

**Fig.1:**
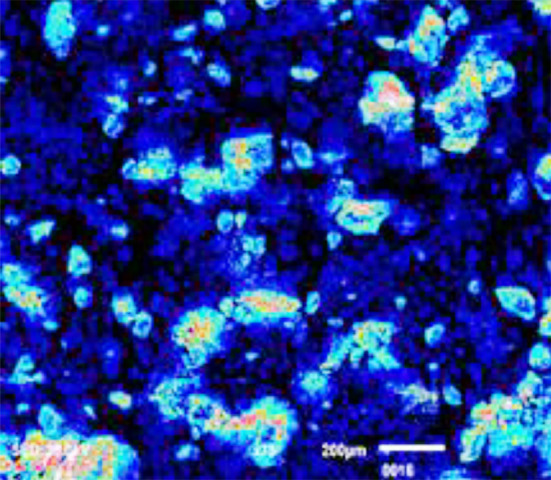
SEM image depicts Chitosan nanoparticles (100nm).

**Fig.2:**
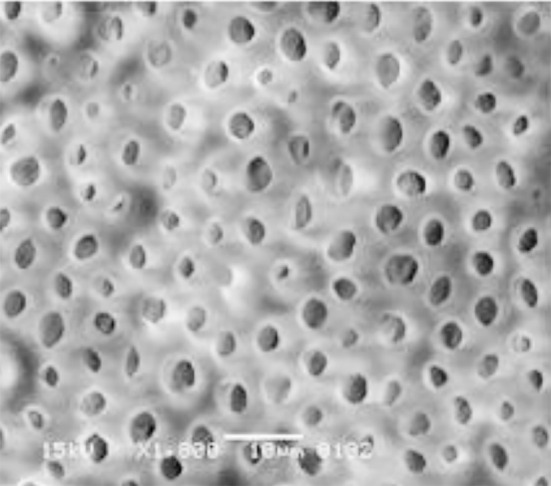
SEM image shows open dentinal tubules with no SL when CHNPs activated via Light activated Irrigation.

**Fig.3:**
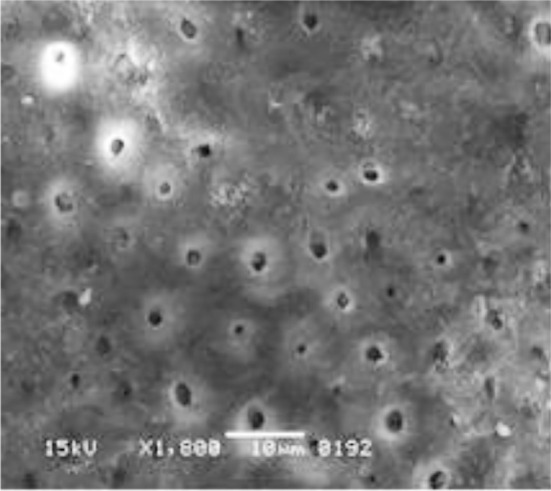
SEM image exhibits closed/narrow dentinal tubules with SL when irrigated with EDTA-SI.

Table-I shows SL removal efficacy after using different final irrigating solutions following two different techniques. Cervical third of Group-IIB (CHNPs-LAI) (1.43±0.27) presented the highest SL removal from the canal dentin. However, apical section of Group-IA (EDTA-SI) (3.55±0.42) samples revealed the lowest SL removal. Comparison among investigated groups demonstrated that Group-IA (Cervical: 2.55±0.18, middle: 2.67±0.32, apical: 3.55±0.42) and Group-IB (CHNPs-SI) (Cervical: 2.43±0.20, middle: 2.60±0.28, apical: 3.41±0.51) revealed no significant difference in the SL efficiency (p>0.05). Likewise, Group-IIA (EDTA-LAI) (Cervical: 1.82±0.38, middle: 1.91±0.34 and apical: 2.01 ±0.46) and Group-IIB (Cervical: 1.43±0.27, middle: 1.56±0.39 and apical: 1.77±0.18) unveiled significantly different outcomes from one another and from another tested group (p<0.05). Intragroup comparison analysis discovered that in Group-IA and Group-IB the cervical and middle sections of the root displayed significantly higher SL removing efficiency than the apical third of the root (p>0.05) Whereas, in Group-IIA and Group-IIB all sections presented comparable SL elimination (p<0.05) (Fig.1-3)

**Table-I:** SL elimination from the canal after using different irrigating solutions and techniques.

Tested groups	Group-IA: EDTA (SI)	Group-IB: CHNPs (SI)	Group-IIA: EDTA (LAI)	Group-IIB: CHNPs (LAI)
Cervical	2.55±0.18 a,C	2.43±0.20 a,C	1.82±0.38 a,B	1.43±0.2 a,A
Middle	2.67±0.32 a,C	2.60±0.28 a,C	1.91±0.34 a,B	1.56±0.39 a,A
Apical	3.55±0.42 b, C	3.41±0.51 b, C	2.01 ±0.46 a,B	1.77±0.18 a,A

ANOVA p<0.05; Sodium hypochlorite (NaOCl), Ethylenediamine-tetraacetic acid (EDTA), Chitosan nanoparticles (CHNPS), Syringe irrigation (SI), Laser activated irrigation (LAI); Distinct superscript lowercase letters within the same column denote statistically significant variations (p <0.05). Distinct uppercase letters within a specific row signify significant differences Post Hoc Tukey’s (p<0.05)

### PBS testing:

Table-II shows the PBS of AH plus sealer to canal dentin after using different irrigating solutions and techniques. Cervical third of Group-IIB (CHNPs-LAI) (12.36±0.56 MPa) presented the maximum bond integrity with the canal dentin. However, apical section of Group-IA (EDTA-SI) (6.43±0.42 MPa) samples revealed the minimum POBS. Comparison among investigated groups discovered that Group-IA (Cervical: 8.35±0.17 MPa, middle:8.20±0.12 MPa, apical: 6.43±0.42 MPa) and Group-IB (CHNPs-SI) (Cervical: 8.41±0.12 MPa, middle: 8.26±0.25 MPa, apical: 6.67±0.36 MPa) disinfected teeth revealed comparable bond scores.(p>0.05) On the other hand, Group-IIA (EDTA-LAI) (Cervical: 10.48±0.41 MPa, middle: 10.21±0.25 MPa and apical: 9.90±0.11 MPa) and Group-IIB (Cervical: 12.36±0.56 MPa, middle: 11.98±0.11 MPa and apical: 11.68±0.13 MPa) unveiled considerably different outcomes from one another and also from other tested group. Comparison within the different sections of the same group discovered that in Group-IA and Group-IB the cervical and middle sections of the root displayed significantly different POBS than the apical third of the root. Whereas, in Group-IIA and Group-IIB all sections presented comparable bond strength scores. (p>0.05).

**Table-II:** PBS of resin-based sealer to canal dentin after using different irrigating solutions and techniques.

Tested groups	Group-IA: EDTA (SI)	Group-IB: CHNPs (SI)	Group-IIA: EDTA (LAI)	Group-IIB: CHNPs (LAI)	p-value^+^
Cervical	8.35±0.17 a,C	8.41±0.12 a,C	10.48±0.41 a,B	12.36±0.56 a,A	(p<0.05)
Middle	8.20±0.12 a,C	8.26±0.25 a,C	10.21±0.25 a,B	11.98±0.11 a,A	
Apical	6.43±0.42 b, C	6.67±0.36 b,C	9.90±0.11 a,B	11.68±0.13 a,A	

+ ANOVA Sodium hypochlorite (NaOCl), Ethylenediamine-tetra acetic acid (EDTA), Chitosan nanoparticles (CHNPS), Syringe irrigation (SI), Laser activated irrigation (LAI); Distinct superscript lowercase letters within the same column denote statistically significant variations (p<0.05). Distinct uppercase letters within a specific row signify significant differences Post Hoc Tukey’s (p<0.05)

### Failure mode assessment:

**Fig.4:**
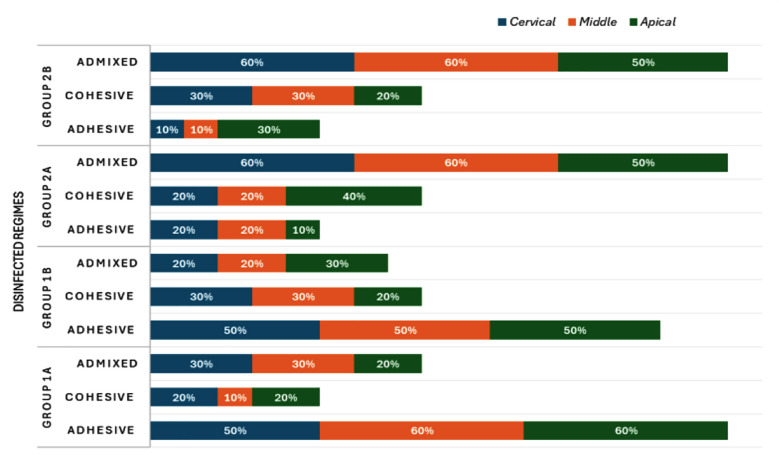
Percentage of failure modes in each investigated group.

In Fig.4, the percentage of failure modes among each investigated group. Group-IA and 1B presented the most adhesive failures. Whereas Group-IIA and 2B exhibited admixed failures predominantly.

## DISCUSSION

The current investigation was predicated on the hypothesis that no statistically significant difference exists in the eradication of SL and the POBS of root canal sealer disinfected with CHNPs when compared to the control EDTA, provided that a similar irrigation technique is employed. Additionally, it was also hypothesized that there would be no significant difference in SL elimination and POBS of the endodontic sealer when disinfection is carried out utilizing LAI technique as opposed to the traditional SI. Based on the results obtained, EDTA and 0.2% CHNPs solutions were identified as the final irrigant, exhibiting similar efficacy in terms of SL efficacy and POBS when applied via SI only, thereby partially validating the initially proposed hypothesis. Additionally, it was determined that LAI-treated groups yielded superior SL elimination and POBS results in contrast to the traditional SI counterpart, thereby completely refuting the second hypothesis.

The results from the present inquiry indicated that a solution of 0.2% CHNPs is comparably effective as a 17% EDTA solution in the elimination of the SL from the canal wall. These findings align with laboratory-based studies conducted by Ratih and associates. They reported that final irrigation with either solution seems to effectively dissolve the SL, particularly its inorganic constituents, albeit through distinct mechanisms.^14^ EDTA acts as a chelating agent by binding to calcium ions present in dentin, which leads to the demineralization of the dentin structure. It decalcifies the dentin up to a depth ranging from 20 to 50 μm within a timeframe of 2-3 minutes.^15^ In a similar vein, chitosan also displays chelating characteristics.

Although the precise nature of its interaction with dentin remains incompletely elucidated, it is posited that adsorption, ion exchange, and chelation play significant roles in the interaction between chelating agents and metal ions.^16^ Moreover, factors such as ionic activity, the chemical composition of chitosan, and the pH of the solution may impact the nature of these interactions.^17^ Two primary hypotheses have been suggested to elucidate the chelating mechanism of chitosan. The first, referred to as the “chemical chain bridge model,” posits that multiple amino groups distributed along the chitosan chain interact with the same metal ion.^18^ The second, called the “hook or free-arm model,” theorizes that only a single amino group participates in the interaction, with the metal ion binding directly to that specific group.^19^

Chitosan is a polymer constituted of repeating chitin dimers, each comprising two nitrogen atoms that possess lone pairs of electrons, thereby facilitating interactions with metal ions. Under acidic environments, the amino groups of this biopolymer undergo protonation (-NH_3_^+^), which facilitates the extraction of additional molecules for adsorption into the root canal dentin, thereby promoting deeper penetration into the dentinal tubules.^20^ The efficacy of SL removal substantiates the positive outcomes observed with POBS, as it fosters improved infiltration of the endodontic sealer into the canal tubules.

LAI, in contrast to the SI, demonstrated markedly enhanced efficacy in the removal of SL and the enhancement of POBS of root canal sealer to the canal dentin. This observation can be elucidated by the phenomenon that the activation of the laser at sub-ablative parameters in a liquid medium result in the generation of substantial vapor bubbles that can expand up to 1600 times their initial volume before collapsing, thereby inducing secondary cavitation effects.^21^ Such dynamics initiate shock waves that subsequently dislodge the SL and disrupt bacterial biofilms. As the laser persists in delivering energy, the light penetrates the bubble and vaporizes the water surface at the forefront of the bubble. Consequently, it creates a conduit through the liquid until the pulse concludes, approximately 140 μs later.^22^ This established mechanism is commonly referred to as the Moses effect within the microsecond timeframe. There is a significant difference exist in the EDTA and CHNPs solution efficiency as a final irrigant when the LAI technique was implemented. The authors of the present study predicted that CHNPs are smaller in size and show lower sessile contact angles, indicating better fluid dynamics within the root canal when activated by laser.^23^

The findings of the current investigation also indicated that LAI groups exhibited no statistically significant difference in SL removal from the apical third relative to the middle and cervical sections of the root. This agrees with the findings of an invitro based investigation conducted by Lei et al.^24^ Syringe Irrigation without activation leads to increased SL scores, especially in the apical third of the canal. Notwithstanding the drawbacks of SI, it continues to be a commonly utilized technique owing to its ease of use and widespread availability.^25^ Nevertheless, the findings indicate that the integration of advanced irrigation methods can significantly improve the cleaning efficacy in endodontic procedures.

### Clinical innovation and Application:

The combination of light-activated irrigation and chitosan represents an emerging approach in endodontic therapy. This innovation addresses the limitations of traditional irrigation methods by combining the chelating properties of chitosan with the enhanced activation provided by light energy, potentially improving both smear layer removal and root canal disinfection in a single treatment protocol.

### Limitations:

The current investigation revealed certain limitations. The research was limited to a laboratory-based trial; hence, care must be taken when extrapolating the findings to a clinical context. Another constraint of this research is the absence of microbial evaluation, which could be performed using Confocal laser scanning Microscopy (CLSM). Simultaneous assessment of SEM and CLSM presents a benefit, as it facilitates an understanding of the relationship between smear layer removal and the inhibition of microbial growth. Additionally, only a single concentration of CHNP was employed, which may have influenced the results.

## CONCLUSION

Chitosan nanoparticles activated with Er: YAG laser as a final root canal sterilant presented better SL removal and push-out bond strength of AH plus sealer to the canal dentin.
